# Medical students' and facilitators' experiences of an Early Professional Contact course: Active and motivated students, strained facilitators

**DOI:** 10.1186/1472-6920-8-56

**Published:** 2008-12-02

**Authors:** Bernhard von Below, Gunilla Hellquist, Stig Rödjer, Ronny Gunnarsson, Cecilia Björkelund, Mats Wahlqvist

**Affiliations:** 1 Department of Public Health and Community Medicine/Primary Health Care, The Sahlgrenska Academy at University of Gothenburg, PO Box 454, SE-405 30 Gothenburg, Sweden; 2 Floda Primary Health Care Center, Southern Älvsborg County, Rurik Holms väg, S-448 30 Floda, Sweden; 3 Section of Hematology, Department of Medicine, Sahlgrenska University Hospital, S-413 45 Gothenburg, Sweden; 4 Research and Development Unit, Primary Health Care, Southern Älvsborg County, Sven Eriksonsplatsen 4, plan 2, S-503 38 Borås, Sweden

## Abstract

**Background:**

Today, medical students are introduced to patient contact, communication skills, and clinical examination in the preclinical years of the curriculum with the purpose of gaining clinical experience. These courses are often evaluated from the student perspective. Reports with an additional emphasis on the facilitator perspective are scarce. According to constructive alignment, an influential concept from research in higher education, the learning climate between students and teachers is also of great importance. In this paper, we approach the learning climate by studying both students' and facilitators' course experiences.

In 2001, a new "Early Professional Contact" longitudinal strand through term 1–4, was introduced at the Sahlgrenska Academy, University of Gothenburg, Sweden. General practitioners and hospital specialists were facilitators.

The aim of this study was to assess and analyse students' and clinical facilitators' experiences of the Early Professional Contact course and to illuminate facilitators' working conditions.

**Methods:**

Inspired by a Swedish adaptation of the Course Experience Questionnaire, an Early Professional Contact Questionnaire was constructed. In 2003, on the completion of the first longitudinal strand, a student and facilitator version was distributed to 86 students and 21 facilitators. In the analysis, both Chi-square and the Mann-Whitney tests were used.

**Results:**

Sixty students (70%) and 15 facilitators (71%) completed the questionnaire. Both students and facilitators were satisfied with the course. Students reported gaining iiration for their future work as doctors along with increased confidence in meeting patients. They also reported increased motivation for biomedical studies. Differences in attitudes between facilitators and students were found. Facilitators experienced a greater workload, less reasonable demands and less support, than students.

**Conclusion:**

In this project, a new Early Professional Contact course was analysed from both student and facilitator perspectives. The students experienced the course as providing them with a valuable introduction to the physician's professional role in clinical practice. In contrast, course facilitators often experienced a heavy workload and lack of support, despite thorough preparatory education. A possible conflict between the clinical facilitator's task as educator and member of the workplace is suggested. More research is needed on how doctors combine their professional tasks with work as facilitators.

## Background

During the last decade, universities worldwide have introduced medical students to patient contact, communication skills and the clinical examination earlier than before [1-3]. These courses are often entitled "Early Clinical Experience" or "Early Patient Contact" and usually employ general practitioners (GPs) as facilitators [4-9]. At present, curricula in all Swedish medical schools include such courses during the preclinical years of the curriculum. From both student and facilitator perspectives, the need to evaluate these innovations in early medical education is apparent.

A common contemporary view in medical education is that the teacher's task is to activate students in order to learn; to be a facilitator of student learning and to arrange and provide learning opportunities for students [10,11]. This is clearly the case in early clinical experience courses. Here, the facilitator has a central role in involving and encouraging students to learn from encounters with doctors, patients and personnel in health care. Students in early medical education are curious and motivated to learn from clinical practice [12]. Besides focus on student learning conditions, facilitator working conditions and perceptions also call for increased consideration.

Biggs' concept of *constructive alignment *adheres to a learner-centred perspective in higher education and highlights the linkage between learning content, teaching and learning activities, and assessment [13]. The concept also acknowledges the student-teacher relationship. According to Biggs, five main components in university education can be distinguished. In order to accomplish a deep learning approach among students, leading to conceptual change and understanding, these main components should be linked in accordance with each other and act as a system:

1) The curriculum

2) The teaching methods

3) The assessment procedures and methods in result reporting

4) The climate created through interactions with students

5) The institutional climate, rules and procedures

In the present study we approached the overall learning climate created through teachers' interactions with students. The learning climate can be hard to study *per se*. Yet, students' and facilitators' course experiences represent two aspects of a course that contribute to the learning climate. These experiences are accessible for study.

Most articles deal with early clinical introduction from the student perspective [3]. Only a few include the facilitator perspective. According to these studies, general practitioners were engaged in early clinical teaching and the most common facilitator task was to teach students individually [3,14,15]. Factors restraining general practitioners from taking on additional teaching were also identified. Still, a better understanding of clinical facilitators' working conditions is needed.

In this study, we approach the learning climate and report both students' and facilitators' experiences in a new Early Professional Contact course.

### Context of study

During the years 1998–2000, a curriculum evaluation was carried out at the medical school of the Sahlgrenska Academy, University of Gothenburg. Weak areas identified in the evaluation were student introduction to the professional role and student learning of generic skills. A renewal of the medical curriculum was recommended. One of the main outcomes of the evaluation was the development and introduction of a new course, "Early Professional Contact" (EPC). Thus, the first EPC course was launched in 2001 as a result of views from both students and teachers. The aim of the course was to introduce students to clinical practice, physician's professional role, to provide knowledge, skills, and inspiration for future work and to strengthen students' motivation for biomedical studies.

The course was set up as a longitudinal strand of students' learning in clinical practice during the first two preclinical years. Students participated in clinical work together with the physician and his or her staff at the clinic. A group of four students was scheduled to meet a clinical EPC facilitator for a year. Thus, the facilitator had to handle a small group of four students; instead of only one or two which was otherwise the common model. The EPC year 1 comprised eight evenly distributed days on clinical attachments. During EPC year 2, students were relocated to new groups, from primary care to hospital attachments and vice versa and met a new facilitator for a further eight days. Students' clinical experiences occurred at community-based practices (Health centres) when the student group had a general practitioner as a facilitator, and at a hospital when the group was facilitated by a specialist in a discipline other than Family Medicine.

Students also attended a mandatory seminar each term, with special themes such as medical ethics, the patient-doctor encounter, and the role of non-course literature (fiction, novels of well-known authors) in medical education. EPC facilitators were educated and prepared in student-centred, task- and experience-based learning methods and in small groups work. Continuity was ensured in the teacher-learner relationship.

Both general practitioners and other specialists were recruited as facilitators. Half the facilitators were general practitioners and half were clinicians from university and county hospitals. It was stressed that students could learn about the physician's professional role irrespective of discipline. Furthermore, the intention was that this diversity of facilitators would stimulate discussion and reflection among students and teachers. Facilitators were recruited from among interested colleagues with teaching experience. The facilitator assignment was voluntary and without personal financial incentive. However, GP health centres and hospitals were financially supported by the university for taking on EPC student groups, the sum being correspondent with the cost for stand-ins for the facilitators at the clinic.

**Figure 1:**
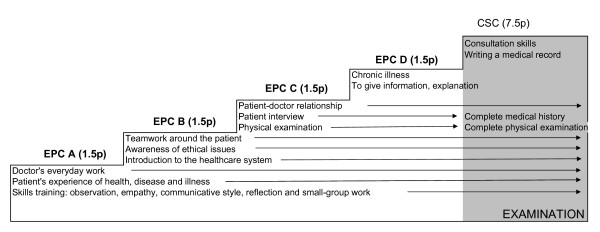
**Progression of EPC learning objectives in a staircase model**. Early Professional Course (EPC) term 1–4, preceding the Consultation skills course, term 5 (CSC).

EPC learning objectives were thoroughly structured to build student competence in a deliberate progression. The EPC course prepared students for the "Consultation skills" course in the fifth term of the curriculum. Performance assessment of student skills was made at the end of the "Consultation skills" course but not during the EPC course (Figure 1). In the first EPC year, the main aims were to provide an orientation of the physician's professional role and of patient experiences of illness and health; to learn about other categories from the staff and their work, organization of health care and basic medical ethics in every-day care. In terms three and four, learning goals included an introduction to patient-centred skills and clinical examination skills. EPC facilitators were the sole source of clinical skills training during these two preclinical years. At course start, each student received a study guide and each facilitator received an expanded teacher version. In the guide, the course's learning goals were presented and aligned with the tasks on each EPC day at the clinic. It also included relevant articles on the focus theme of EPC days and seminars.

Aims of this study were to assess and analyse both students' and clinical facilitators' experiences of the new "Early Professional Contact" course in the Gothenburg undergraduate medical program. A further aim was to illuminate the facilitators' working conditions by analysing and comparing students' and facilitators' course experiences.

## Methods

### Participants and materials

#### Students

There were 86 students attending the fourth semester of the EPC course: 51 women and 35 men. Median age was 24 years (range 20–54).

#### Facilitators

A total of 21 facilitators participated in the first EPC course. Ten were general practitioners while eleven were specialists in other disciplines: internal medicine (five), surgery (two), geriatrics, medical rehabilitation, rheumatology and infectious diseases (one of each). There were fourteen men and seven women with a median age of 55 years (range 41–62). All facilitators were given a four-day education before starting the course. During the two-year course period, one or two facilitator meetings were held each semester.

### Procedures

In May 2003, students and facilitators were given an anonymous questionnaire at a mandatory seminar at the end of the course. They were informed that the survey was part of a research evaluation of the course and that participation was anonymous and voluntary. They were given approximately 20 minutes to complete the questionnaire.

### Questionnaire

#### Measurement of course experience: "Early Professional Contact Questionnaire"

A short form of the questionnaire "Course Experience Questionnaire" (CEQ) by Ainley & Long [16,17] was used regularly in Australia and Great Britain to study student opinion after a course or upon completion of academic studies. The original short form of the CEQ consisted of 25 items studying good teaching, clear goals and standards, generic skills, appropriate assessment, appropriate workloads and course satisfaction. Broomfield and Bligh [18] validated the short form of CEQ as an instrument for use with undergraduate medical students and demonstrated satisfactory construct validity and reliability.

Lander & Larson [19] modified and translated the short form of CEQ into Swedish. Lander expanded the questionnaire but retained the structure. He suggested an additional 15 items and this Swedish 40-item questionnaire was used in 1997 at six faculties of the University of Gothenburg, including the faculty of Medicine.

For the specific purpose of evaluating student course experiences in EPC, a new questionnaire was constructed. This consisted of seven items from CEQ translated by Lander, four of Lander's new items and 17 additional items related to the new course. This new questionnaire was entitled "Early Professional Contact Questionnaire" (EPCQ) and consisted of 28 statements on education, goals, workload, skills and general satisfaction (Table 1).

**Table 1:** Early Professional Contact Questionnaire

**Item**^**a**^	**Students' EPCQ**	**Facilitators' EPCQ**
1	Seminars were stimulating and provided valuable knowledge	Seminars were stimulating and provided valuable knowledge
2	After this year, I feel increased confidence when meeting patients	After this year, my students feel increased confidence when meeting patients
3	The workload has been too heavy in this course	My workload as a facilitator has been too heavy in this course
4	My facilitator has stimulated me to contribute with my own experiences, thoughts and knowledge	I have stimulated students to contribute with their own experiences, thoughts and knowledge
5	The demands on me have been reasonable on this course	The demands on me as a facilitator have been reasonable on this course
6	It was often hard to discover what I was expected to learn during this course	It was often hard to discover what I was expected to teach during this course
7	Our group talks has increased my understanding of what I have participated in	Our group talks has increased students' understanding of what they have participated in
8	The structure of the EPC days has been good	The structure of the EPC days has been good
9	The tasks during the EPC days have been important for my learning	The tasks during the EPC days have been important for students learning
10	I have practiced my perceiving and understanding of patients' feelings	Students have practiced their perceiving and understanding of patients' feelings
11	The course has given me valuable insight into medical profession and medical service	The course has given students valuable insight into medical profession and medical service
12	We students have had the opportunity to influence the content of the EPC days	Students have had the opportunity to influence the content of the EPC days
13	My facilitator has given me helpful feed-back in practical situations	I have given students helpful feedback in practical situations
14	The course has increased my motivation for biomedical studies	My colleagues have taken interest in my students
15	I have trained observing patients' general health condition	Students have trained observing patients' general health condition
16	My facilitator has listened to us students, has been taking us seriously and reacting	I have listened to the students, have been taking them seriously and reacting
17	Course leaders have given us sufficient information	Course leaders have given me information and support
18	The course has inspired me to the medical profession	The course has inspired me for coming tasks as a facilitator
19	The study guide has been useful for my studies during this course	The study guide has been useful in planning and influencing the practice days
20	My facilitator has worked hard to make the course interesting	I have worked hard to make my teaching interesting
21	I have enjoyed this course	I have got pleasure out of my task as a facilitator
22	The discussions during seminars have been fruitful	The discussions during seminars have been fruitful
23	My facilitator has encouraged me in a number of ways	My head of the clinic has encouraged me in a number of ways in my work as a facilitator
24	EPC has been an interesting and valuable course	EPC has been an interesting and valuable course
25	I have trained my understanding of people's experience of health and illness	Students have been trained in understanding people's experience of health and illness
26	My small student group has functioned well	I have had a well functioning student group
27	The aim of the course EPC has been fulfilled	The aim of the course EPC has been fulfilled
28	In general, I am satisfied with the quality of this course	Facilitator education and facilitator meetings have given me a good and firm ground as facilitator

^a ^The origin of items were as follows: Item 3, 5, 6, 13, 20, 23 and 28 came from short form of Course Experience Questionnaire (CEQ) and were translated to Swedish by Lander. Items 4, 12, 16 and 24 were developed by Lander et al. Items 1, 2, 7–11, 14, 15, 17–19, 21, 22 and 25–27 were developed specifically for Early Professional Contact Questionnaire (EPCQ).

The participants agreed or disagreed using a five-degree, Likert scale anchored by (1) Agree and (5) Disagree.

A facilitator version of the questionnaire was constructed (Table 1). The participants agreed or disagreed using a five-degree, Likert scale anchored by (1) Agree and (5) Disagree.

Discussing each item thoroughly with teachers, facilitators, and researchers at the university ensured face validity of the EPCQ.

### Ethical aspects

Before the onset of the study, ethical issues concerning the design of the study were considered. Subject participation in answering the questionnaire was voluntary and anonymous as a normal part of course evaluation. Questions covered students' and facilitators' ordinary education activities but not sensitive or personal matters. The potential negative influence on subjects from answering the questionnaire was judged as negligible. The study was reviewed by the Programme Board of Medical Education and representatives of the Medical Students Organisation. These ethical considerations were judged as corresponding to requisite ethical review. According to Swedish legislation regarding ethical vetting on research involving human beings, an ethical evaluation by a regional ethical review board was not required.

### Statistical methods

#### Analysis and comparison of students' and facilitators' course experiences

Items were categorized in three classes. In the first class, denoted A and containing 18 items, student and facilitator content were identical or nearly identical. In the next class, denoted B and containing five items, non-identical but similar aspects of the course were covered. In the third class, denoted C and containing five items, different aspects between students and facilitators were studied. Mean and median was calculated for all items. Further analysis depended on class.

In items of class A, the focus was on the association between facilitator and student experience. Since data was measured on an ordinal scale, Spearman's rank correlation was considered suitable. However, since questionnaires were anonymous, facilitators and students could not be matched. Thus, Spearman's rank correlation could not be used. As a measure of association chi-square in a 5 × 5 table was calculated and chi-square was transformed for each item to both a two-tailed p-value and to Cramer's V-index. Thus, the V-index was an effect size measure of association and the p-value indicated if the V-index was statistically significant.

In items of class B the focus was on differences in experience between students and facilitators. Due to data being measured on an ordinal scale the statistical significance was estimated by the two-tailed Mann-Whitney's test and presentation of each group's median value was used as an nonstandardised effect size measure.

In items of class C there was no relevance in comparing facilitators and students whereby these items evaluated different aspects among students and facilitators.

P-values ≤0.05 were considered statistically significant. The computer programme Epi Info version 3.2.2 (CDC, Atlanta) was used for the statistical analysis.

## Results

### Students' course experiences

Of 86 students, 60 (70%) completed the questionnaire (Table 2).

**Table 2:** Outcome of Students' Early Professional Contact Questionnaire (EPCQ)

			**Agree**			**Disagree**	
*Item*	*Aspect*	*Question*	**1**	**2**	**3**	**4**	**5**
1	**Seminars**	Seminars were stimulating and provided valuable knowledge	8	26	17	7	2
2	**Confidence**	After this year, I feel increased confidence when meeting patients	26	26	6	2	0
3	**Workload**	The workload has been too heavy in this course	0	3	9	13	35
4	**Students contribution**	My facilitator has stimulated me to contribute with my own experiences, thoughts and knowledge	27	24	7	2	0
5	**Demands**	The demands on me have been reasonable on this course	37	19	3	1	0
6	**Expectations**	It was often hard to discover what I was expected to learn during this course	7	18	15	13	7
7	**Group talks**	Our group talks has increased my understanding of what I have participated in	17	23	12	7	1
8	**EPC day structure**	The structure of the EPC days has been good	6	37	11	6	0
9	**EPC day tasks**	The tasks during EPC days have been important for my learning	13	19	18	8	2
10	**Patients feelings**	I have practiced my perceiving and understanding of patients' feelings	17	22	15	6	0
11	**Medical profession**	The course has given me valuable insight into medical profession and medical service	40	18	2	0	0
12	**Students influence**	We students have had the opportunity to influence the content of EPC days	23	26	8	3	0
13	**Feedback**	My facilitator has given me helpful feed-back in practical situations	19	20	13	5	3
14	**Biomedical study motivation**	The course has increased my motivation for biomedical studies	23	24	9	2	2
15	**Clinical skill**	I have trained observing patients' general health condition	8	26	14	8	4
16	**Listening to students**	My facilitator has listened to us students, has been taking us seriously and reacting	36	15	8	1	0
17	**Course leaders**	Course leaders have given us sufficient information	19	23	16	2	0
18	**Inspiration**	The course has inspired me to the medical profession	37	15	7	1	0
19	**Study guide**	The study guide has been useful for my studies during this course	0	1	14	15	30
20	**Facilitators' effort**	My facilitator has worked hard to make the course interesting	31	21	2	6	0
21	**Enjoyed course**	I have enjoyed this course	16	29	11	4	0
22	**Seminars**	The discussions during seminars have been fruitful	5	24	22	4	5
23	**Encouragement**	My facilitator has encouraged me in a number of ways	17	26	14	2	1
24	**Interesting course**	EPC has been an interesting and valuable course	29	21	7	3	0
25	**Training understanding experience**	I have trained my understanding of people's experience of health and illness	24	26	7	2	1
26	**Student group**	My small student group has functioned well	39	16	2	1	2
27	**Course aim**	The aim of the course EPC has been fulfilled	13	35	11	0	1
28	**Course quality**	In general, I am satisfied with the quality of this course	28	25	5	1	1

The students found the course interesting and beneficial. They reported becoming inspired toward their future work as doctors and felt increased confidence when meeting patients. In addition, the EPC course provided increased motivation for their parallel biomedical studies. Students reported that their facilitator stimulated them to contribute with their own experiences and knowledge and were given an opportunity to influence the content of the EPC days.

### Facilitators' course experiences

Of the 21 facilitators, 15 (71%) completed the questionnaire (Table 3). They found the course interesting and rewarding and took pleasure in the task of facilitator. They also reported that the EPC course inspired them to continue work as facilitators. Furthermore, the facilitators reported that their students were trained in perceiving and understanding their patient's feelings and in observing the patient's general health condition and that their students gained increased confidence when meeting patients while obtaining an orientation to the medical profession. Facilitator preparatory education and facilitator meetings were highly appreciated. However, despite efforts to promote clinical teaching as an important assignment, facilitators experienced that they lacked support from superiors at the clinic.

**Table 3:** Outcome of Facilitators' Early Professional Contact Questionnaire (EPCQ)

			**Agree 1**		**Disagree 5**		
*Item*	*Aspect*	*Question*	**1**	**2**	**3**	**4**	**5**
1	**Seminars**	Seminars were stimulating and provided valuable knowledge	5	8	2	0	0
2	**Students' confidence**	After this year, my students feel increased confidence when meeting patients	8	7	0	0	0
3	**Workload**	The workload as a facilitator has been too heavy in this course	1	4	6	3	1
4	**Students contribution**	I have stimulated students to contribute with their own experiences, thoughts and knowledge	2	12	1	0	0
5	**Demands**	The demands on me as a facilitator have been reasonable on this course	4	9	1	1	0
6	**Expectations**	It was often hard to discover what I was expected to teach during this course	2	3	1	4	5
7	**Group talks**	Our group talks has increased students' understanding of what they have participated in	5	9	1	0	0
8	**EPC day structure**	The structure of the EPC days has been good	5	7	2	1	0
9	**EPC day tasks**	The tasks during EPC days have been important for students learning	5	7	2	1	0
10	**Patients feelings**	Students have practiced their perceiving and understanding of patients' feelings	4	11	0	0	0
11	**Medical profession**	The course has given students valuable insight into medical profession and medical service	10	3	2	0	0
12	**Students' influence**	Students have had the opportunity to influence the content of practice days	4	10	1	0	0
13	**Feedback**	I have given students helpful feedback in practical situations	4	9	2	0	0
14	**Colleagues**	My colleagues have taken interest in my students	6	3	5	1	0
15	**Clinical skill**	Students have trained observing patients' general health condition	2	12	1	0	0
16	**Listening to students**	I have listened to the students, have been taking them seriously and reacting	8	7	0	0	0
17	**Course leaders**	Course leaders have given me information and support	8	5	2	0	0
18	**Inspiration**	The course has inspired me for coming tasks as a facilitator	7	5	1	2	0
19	**Study guide**	The study guide has been useful in planning and effecting the EPC days	9	3	2	1	0
20	**Teaching effort**	I have worked hard to make my teaching interesting	12	2	1	0	0
21	**Pleasure**	I have got pleasure out of my task as a facilitator	9	3	1	2	0
22	**Seminars**	The discussions during seminars have been fruitful	5	7	2	1	0
23	**Encouragement**	My head of the clinic has encouraged me in a number of ways in my work as a facilitator	3	1	9	1	1
24	**Interesting course**	EPC has been an interesting and valuable course	9	5	1	0	0
25	**Health experience**	Students have been trained in understanding people's experience of health and illness	7	8	0	0	0
26	**Student group**	I have had a well functioning student group	10	3	2	0	0
27	**Course aim**	The aim of the course EPC has been fulfilled	5	7	2	1	0
28	**Facilitator education**	Facilitator education and facilitator meetings have given me a good and firm ground as facilitator	9	5	1	0	0

### Analysis of both students' and facilitators' course experiences

The association of students' and facilitators' course experiences is depicted in Table 4. Both facilitators and students agreed that students were stimulated to contribute with individual experiences, thoughts and knowledge (p = 0.048) (item 4, Table 4). Furthermore they agreed that students had practiced perceiving and understanding patient feelings (p = 0.03) (item 10, table 4).

**Table 4:** Asssociation and comparison of student and facilitator perspectives in EPCQ

		Students		Facilitators		Statistical evaluation – students and facilitators		
Item	Class^a^	Mean^b^	Median^c^	Mean^b^	Median^c^	Type of analysis	Cramer's V^d^	P-value^e^
1	A	2.5 (0.98)	2 (2–3)	1.8 (0.68)	2 (1–2)	Association of stimulating seminars	0.29	0.17
2	A	1.7 (0.78)	2 (1–2)	1.5 (0.52)	1 (1–2)	Association of increased students' confidence	0.17	0.51
3	B	4.3 (0.91)	5 (4–5)	2.9 (1.0)	3 (2–4)	Comparison of experienced workload in the course	-----	<0.0001
4	A	1.7 (0.80)	2 (1–2)	1.9 (0.46)	2 (2–2)	Association of students' contribution	0.32	0.048
5	B	1.5 (0.68)	1 (1–2)	1,9 (0,80)	2(1–2)	Comparison of experienced demands on this course	-----	0.017
6	B	2.9 (1.2)	3 (2–4)	3.5 (1,5)	4 (2–5)	Comparison of expectations during this course	-----	0.15
7	A	2.2 (1.0)	2 (1–3)	1.7 (0.59)	2 (1–2)	Association of view on group talks	0.25	0.32
8	A	2.2 (0.78)	2 (2–3)	1.9 (0.88)	2 (1–2)	Association of EPC day structure	0.26	0.15
9	A	2.5 (1.1)	2 (2–3)	1.9 (0.88)	2 (1–2)	Association of EPC day tasks	0.22	0.45
10	A	2.2 (0.96)	2 (1–3)	1.7 (0.46)	2 (1–2)	Association of students understanding patients' feelings	0.34	0.030
11	A	1.4 (0.55)	1 (1–2)	1.5 (0.74)	1 (1–2)	Association of insight into medical profession	0.19	0.26
12	A	1.9 (0.84)	2 (1–2)	1.8 (0.56)	2 (1–2)	Association of students' influence	0.20	0.39
13	A	2.2 (1.1)	2 (1–3)	1.9 (0.64)	2 (1–2)	Association of feedback	0.25	0.31
14	C	1.9 (0.99)	2 (1–2)	2.1 (1.0)	2 (1–3)	-----	-----	-----
15	A	2.6 (1.1)	2 (2–3)	1.9 (0.46)	2 (2–2)	Association of training clinical skills	0.32	0.097
16	A	1.6 (0.85)	1 (1–2)	1.5 (0.52)	1 (1–2)	Association of listening to students	0.24	0.23
17	C	2.0 (0.85)	2 (1–3)	1.6 (0.74)	1 (1–2)	-----	-----	-----
18	C	1.5 (0.77)	1 (1–2)	1.9 (1.1)	2 (1–2)	-----	-----	-----
19	B	4,2 (0,87)	4.5 (3,5-5)	1,7 (0,98)	1 (1–2)	Comparison of usefulness of study guide	-----	<0.0001
20	A	1.7 (0.94)	1 (1–2)	1.3 (0.59)	1 (1–1)	Association of facilitators' effort	0.27	0.14
21	C	2.1 (0.85)	2 (1–2.5)	1.7 (1.1)	1 (1–2)	-----	-----	-----
22	A	2.7 (1.0)	3 (2–3)	1.9 (0.88)	2 (1–2)	Association of fruitful seminars	0.35	0.06
23	B	2,1 (0,90)	2 (1–3)	2,7 (1,1)	3 (2–3)	Comparison of encouragement from others	-----	0.016
24	A	1.7 (0.86)	2 (1–2)	1.5 (0.64)	1 (1–2)	Association of finding the course interesting	0.13	0.71
25	A	1.8 (0.87)	2 (1–2)	1.5 (0.52)	2 (1–2)	Association of training health experience	0.20	0.57
26	A	1.5 (0.91)	1 (1–2)	1.5 (0.74)	1 (1–2)	Association of well functioning small student group	0.21	0.52
27	A	2.0 (0.74)	2 (2–2)	1.9 (0.88)	2 (1–2)	Association of fulfilled course aim	0.27	0.25
28	C	1.7 (0.83)	2 (1–2)	1.5 (0.64)	1 (1–2)	-----	-----	-----

^a ^A = Students' and facilitators' items concerns identical or nearly identical aspects thus making statistical estimation of association with Cramer's V possible. B = Students and facilitators items estimated the same aspect but for themselves (for example tutor's workload versus student's workload) making comparison with Mann-Whitney's test possible. C = Students and facilitators items estimated different aspects making estimation of association and comparison unsuitable.

^b ^Mean (Standard deviation)

^c ^Median (Interquartile range)

^d ^For items of class A (identical items) association between students and facilitators response was estimated by Cramer's V index.

^e ^For items in class A p-value is based on chi-square to estimate if the corresponding Cramer's V is of interest. For items in class B (similar items) p-value represents comparison of students and facilitators with Mann-Whitney's test.

Differences in course experiences between facilitators and students (table 4) showed that facilitators experienced a greater workload (item 3), less reasonable demands (item 5) and less encouragement than the students (item 23) and found the study guide much more useful than the students (item 19).

## Discussion

The Early Professional Contact course was the first early clinical introduction course at the Sahlgrenska Academy. According to evaluations of the EPC Course Questionnaire, both students and facilitators were satisfied with the course. The students found the course interesting and beneficial. They reported increased confidence when meeting patients and were inspired to their future work as doctors. Facilitators experienced a greater workload, less reasonable demands and less support than students. Thus, a discrepancy was observed.

### Methodological considerations

Among students, participation rate was 70% and among facilitators 71%. This was regarded as acceptable for students but somewhat tenuous for facilitators as their total number was only 21.

Student representatives expressed worries about registering student views. Thus, we considered anonymity of the highest priority. Consequently, it was not possible to trace, record or analyse non-responders.

The Early Professional Contact Questionnaire was constructed as a combination of new questions with questions from the translation of the original CEQ and from Lander's new questions as described above. The aim was to create a questionnaire feasible and practical for this new course evaluation. However, deconstructing the original CEQ and Lander's questionnaire invalidates the prior validation of these methods. Discussing each item thoroughly with teachers, facilitators, and researchers at the university ensured face validity of the EPCQ. However, further research should investigate criterion validity of EPCQ by estimating its association with the outcome of similar questionnaires.

We have discussed the relevance of comparing results of items where students and facilitators estimated the same course aspect separately (the five items 3, 5, 6, 19, 23) However, these items concerned aspects of the course of interest to compare since course experiences associated with these central aspects contributed to the overall learning environment. An example of this is facilitator's workload versus student's workload. Yet, there was no relevance in comparing those items that reflected fundamentally different aspects of student and teacher tasks in education (items 14, 17, 18, 21 and 28).

The alpha-level of statistical significance was not adjusted for multiple testing. Thus, p-values between 0.01–0.05 should be interpreted with caution.

Cramer's V-index was used as a measure of effect size for items of class A. For class B variables ordered as categorical variables without equidistance we refrained from constructing a conventional measure of effect size. To avoid subtraction we prefer to present median values for each group (Table 4) without performing further mathematical procedures.

### Comments on results

Results from the EPC questionnaire demonstrated that the aims of the course had been met from the students' point of view. Learning goals were to introduce medical students to clinical practice and physician's professional development. A major concern was to provide students with access to clinical experiences and participation in physician's clinical work in a safe and non-judging environment during the theoretical preclinical phase. When starting the EPC course, course leaders were familiar with learning goals and assessment conducted by colleagues in the Consultation skills course in term 5. Due to these circumstances, no summarizing assessment of learning goals in terms of student performance was arranged in the EPC course. However, EPC students' participation was mandatory and was registered by their facilitators. Over the years, a longitudinal learning progression was created as the EPC course was followed by the Consultation Skills course of term five (Figure 1).

The EPC course also increased student motivation for biomedical studies. This result is interesting since objections were raised that early professional contact would distract student attention from biomedical studies. On the contrary, it seems that EPC facilitated an interest in biomedical studies by connecting theory to clinical practice. Positive effects of integrating courses similar to EPC in the curriculum of undergraduate medical studies have been reported previously [3,6,12,20-23]. Our results confirm the value of early clinical introduction as a means of integrating clinical experience in the preclinical basic science phase of a traditional medical curriculum.

Facilitators found the study guide useful but the students did not. One explanation for this may be that the preclinical students, who normally attended lectures and read large textbooks in basic sciences in the EPC course, were now focused on clinical and practical training. Another contributing factor is the lack of assessment, thus affecting student external motivation [11]. Furthermore, it is possible that students were restricted from using the study guide by heavy workloads in parallel biomedical courses, thus lowering their internal motivation.

Consequently, main student expectation of facilitators was to arrange learning events during the EPC days. However, course leaders noticed a remarkably growing interest in the study guide during following courses. The study guide has been revised after discussions with course leaders, facilitators and students.

Facilitators reported that they had stimulated students to contribute with individual experiences, thoughts and competence. These reports corresponded well to the results from students. Associations between students' and clinical facilitators' course experiences were also seen in items concerning student understanding of patient feelings. However, no association was displayed regarding the central aspect of giving feedback to students (item 13). This might indicate that feedback to students is a difficult task requiring practice [24]. Moreover, feedback needs to be related to and supplemented by clear and attainable learning goals. The issue of feedback was often discussed during facilitator meetings and many expressed a need for more training in how to give feedback.

According to Biggs' concept of *constructive alignment*, the second main component in university education consists of teaching methods. The EPC small group model (four students led by one facilitator) appeared to function well (item 7, 26). According to students' opinion and facilitators' views, sharing experiences of the EPC day by reflection and discussion in small groups was helpful in student learning. However, no statistical associations were found between students' and facilitators' results in these two items.

It was interesting to see that facilitators reported that the workload and demands were high while students found course workloads low. Students still experienced that course goals were met.

The importance of being well prepared, when beginning as a facilitator, is well known [25]. It is important that facilitators are adequately educated, given proper time and that aims, course content and students' own activities and facilitator tasks are well defined and clearly communicated [13]. Overly high ambition among facilitators might widen their perception of main learning goals. Students can also develop a pattern of "consuming" practical learning events. The risk is then apparent that the facilitator might experience a heavy workload. If facilitator workload and demands continue to remain at too high a level, they might find their task overburdening and resign. To avoid this, facilitators should encourage their students to be active and share responsibility for the learning outcome of the EPC days. Students' opinion concerning the study guide might support this. Moreover, in this early phase of medical education, students may feel insecure and bewildered by all the new experiences in clinical practice [26]. This may also result in a tendency to cling to their facilitators. Finding the right balance between challenge and support is a well-known dilemma in student-teacher relationships [27]. Continuous contact between facilitators and course leaders is also important and regular facilitator meetings should be held. However, it must also be mentioned that in this study, facilitators found the course interesting and fruitful and reported EPC as having inspired them to continue their assignment.

There was a significant difference between students' and facilitators' experiences of support. The majority of students felt their EPC facilitator supported them. The facilitators, however, felt much less support from their heads of the clinics. This is a serious problem when accepting the role of facilitator and has been previously noted [28]. It was also discussed during the regularly held facilitator meetings. Some facilitators reported problems with heads not supporting or accepting their task as facilitators. This problem could be illuminated by returning to Biggs' *constructive alignment*, in which institutional climate, rules and procedures represent the fifth main component in university education. It is vital that faculty working as clinical facilitators have acceptance and support for their task from their heads and colleagues. This is necessary in order to enable the doctor to engage in educating future physicians. Therefore, our result that the support from facilitators' heads of clinics was low should be taken very seriously. In many instances in health care today, short-term perspectives on economic effectiveness appear to have taken the upper hand. Thus, the individual facilitator is left to handle a conflict between "health care production" and the reproduction of professional knowledge and skills to future colleagues; medical students' need of an education in the clinical context. The central question of student access to clinical learning experiences and facilitators appears to have been neglected on a system level, at least in the Swedish context. Helping medical students to learn in practice should be recognized and included as an important long-term task for the health care system. Providing a positive learning environment for medical students demands much more attention in negotiations between the academy, the medical profession, and health authorities.

By studying the experiences of doctors as clinical facilitators we approach how doctors combine clinical work with their task as facilitators. Knowledge of the physician's perspective as a facilitator might provide the means to increase the positive experience of being a facilitator, thus assisting recruitment. The literature in this research area is growing and expanding [29-31]. Facilitator working conditions are likely to affect student learning. In this EPC course, the assignment as facilitator was voluntary, which likely resulted in motivated and interested facilitators. On the other hand, recruiting facilitators on a voluntary basis can be very time consuming and unpredictable. At many medical schools, the task of facilitator is compulsory and included in a physician's duties. Such a model might provide more stability but also addresses the need for further research on how to combine patients and students in daily medical work.

## Conclusion

Both students and facilitators appreciated this new "Early Professional Course" at the medical school at the Sahlgrenska Academy, Gothenburg, Sweden. According to students' evaluations, they have gained a valuable introduction to the physician's professional role in clinical practice. Students' motivation for biomedical studies was also strengthened. We found differences in course experiences between clinical facilitators and students regarding some aspects of this EPC course. The facilitators reported greater workload, less reasonable demands and less support than the students. Good courses need good facilitators. It seems important that facilitators are well educated and prepared for their task and are provided with adequate support, time and encouragement from heads of clinics and colleagues.

## Competing interests

The authors declare that they have no competing interests.

## Authors' contributions

BB, SR and GH have made substantial contributions to conception, design, acquisition and analysis of data and have been involved in drafting the manuscript. RG and MW have made substantial contributions to analysis and interpretation of data, and were involved in drafting the manuscript and revising it critically. CB has participated in analysis and interpretation of data and has been revising the manuscript critically.

All authors read and approved the final manuscript.

## Pre-publication history

The pre-publication history for this paper can be accessed here:


